# Determination of Dehydrogenase Activities Involved in D-Glucose Oxidation in *Gluconobacter* and *Acetobacter* Strains

**DOI:** 10.3389/fmicb.2016.01358

**Published:** 2016-08-30

**Authors:** Florencia Sainz, María Jesús Torija, Minenosuke Matsutani, Naoya Kataoka, Toshiharu Yakushi, Kazunobu Matsushita, Albert Mas

**Affiliations:** ^1^Departament de Bioquímica i Biotecnologia, Facultat d’Enologia, Universitat Rovira i VirgiliTarragona, Spain; ^2^Department of Biological Chemistry, Faculty of Agriculture, Yamaguchi UniversityYamaguchi, Japan

**Keywords:** acetic acid bacteria, D-gluconic acid, keto-D-gluconic acids, strawberry beverage

## Abstract

Acetic acid bacteria (AAB) are known for rapid and incomplete oxidation of an extensively variety of alcohols and carbohydrates, resulting in the accumulation of organic acids as the final products. These oxidative fermentations in AAB are catalyzed by PQQ- or FAD- dependent membrane-bound dehydrogenases. In the present study, the enzyme activity of the membrane-bound dehydrogenases [membrane-bound PQQ-glucose dehydrogenase (mGDH), D-gluconate dehydrogenase (GADH) and membrane-bound glycerol dehydrogenase (GLDH)] involved in the oxidation of D-glucose and D-gluconic acid (GA) was determined in six strains of three different species of AAB (three natural and three type strains). Moreover, the effect of these activities on the production of related metabolites [GA, 2-keto-D-gluconic acid (2KGA) and 5-keto-D-gluconic acid (5KGA)] was analyzed. The natural strains belonging to *Gluconobacter* showed a high mGDH activity and low activity in GADH and GLDH, whereas the *Acetobacter malorum* strain presented low activity in the three enzymes. Nevertheless, no correlation was observed between the activity of these enzymes and the concentration of the corresponding metabolites. In fact, all the tested strains were able to oxidize D-glucose to GA, being maximal at the late exponential phase of the AAB growth (24 h), which coincided with D-glucose exhaustion and the maximum mGDH activity. Instead, only some of the tested strains were capable of producing 2KGA and/or 5KGA. In the case of *Gluconobacter oxydans* strains, no 2KGA production was detected which is related to the absence of GADH activity after 24 h, while in the remaining strains, detection of GADH activity after 24 h resulted in a high accumulation of 2KGA. Therefore, it is possible to choose the best strain depending on the desired product composition. Moreover, the sequences of these genes were used to construct phylogenetic trees. According to the sequence of *gcd*, gene coding for mGDH, *Acetobacter* and *Komagataeibacter* were phylogenetically more closely related each other than with *Gluconobacter*.

## Introduction

Acetic acid bacteria (AAB) are gram-negative, ellipsoidal to rod-shape acidophilic bacteria and are obligate aerobes (De Ley and Swings, 1984; Deppenmeier et al., 2002). These bacteria could occur in sugary natural environments such as fruits, honey bees, or flowers and in artificial and manmade environments such as soft drinks, cider, beer, wine, or vinegar (De Ley and Swings, 1984). AAB are well known for the rapid and incomplete oxidation of a broad range of sugars, sugar alcohols, and sugar acids (such as D-glucose, glycerol, D-sorbitol, ethanol, or D-gluconic acid) resulting in the accumulation of high amounts of the oxidized products in the culture medium (Asai, 1968; Deppenmeier et al., 2002; Elfari et al., 2005). This capacity allows for the use of AAB for a variety of biotechnological processes in which they carry out oxidative fermentation to obtain several useful compounds that are difficult to be prepared with chemical processes or to be produced with high yields (Gupta et al., 2001; Deppenmeier et al., 2002). Some examples of this metabolism are the production of acetic acid from ethanol or D-gluconic acid (GA) from D-glucose (Deppenmeier et al., 2002; Prust et al., 2005; Lino et al., 2012). Most of these oxidative reactions are catalyzed by membrane-bound dehydrogenases, with reactive centers that are oriented to the periplasmic space (Matsushita et al., 1994). This implies that transport of substrates inside the cell is unnecessary and accumulation of oxidized products in the medium is rapid and near-quantitative (Deppenmeier et al., 2002; Adachi et al., 2003; Matsushita et al., 2003; Elfari et al., 2005; Merfort et al., 2006).

In AAB, many membrane-bound oxidoreductases have been described, and most of these oxidoreductases are pyrroloquinoline quinone (PQQ-) or flavin (FAD-) dependent proteins (Saichana et al., 2015). The oxidative reaction with these dehydrogenases results in bioenergy for AAB because electrons extracted from the substrates are transferred via ubiquinone to the terminal ubiquinol oxidase (Adachi et al., 2007). In D-glucose oxidation, several enzymes located on the periplasmic face of the cytoplasmic membrane catalyze D-glucose oxidation sequentially. Membrane-bound PQQ-glucose dehydrogenase (mGDH) oxidizes D-glucose to glucono-δ-lactone, and it is then converted to GA by glucono-δ-lactonase or spontaneously (Matsushita et al., 1994; Shinagawa et al., 1999). Therefore, mGDH is the enzyme responsible for the production of most GA from D-glucose during fermentation (Macauley et al., 2001). GA can be further converted to 2-keto-D-gluconic acid (2KGA) or 5-keto-D-gluconic acid (5KGA) by two different membrane-bound dehydrogenases (Matsushita et al., 1994; Saichana et al., 2015). One protein is D-gluconate dehydrogenase (GADH), which is a FAD-dependent enzyme (flavoprotein-cytochrome *c* complex) reacting with GA as its only substrate and is responsible for the oxidation of GA to 2KGA (Matsushita et al., 1994; Adachi et al., 2007; Toyama et al., 2007). The membrane-bound dehydrogenase involved in the 5KGA production has been unidentified for a long time, and no specific 5KGA-yielding gluconate dehydrogenase has been found in AAB. Instead, it has been shown that this reaction is catalyzed by a glycerol or polyol dehydrogenase (GLDH, membrane-bound glycerol dehydrogenase), which shows a broad substrate specificity toward several sugar alcohols (D-glycerol, D-sorbitol, D-arabitol, or D-mannitol). Therefore, it is concluded that other PQQ-dependent dehydrogenases such as D-arabitol dehydrogenase (ARDH) or D-sorbitol dehydrogenase (SLDH) are identical to GLDH (Matsushita et al., 2003; Adachi et al., 2007). 2KGA could be further oxidized to 2,5-diketo-D-gluconate by the FAD-dependent 2-keto-D-gluconate dehydrogenase (2KGDH), which is characterized as a flavoprotein-cytochrome *c* complex with three different subunits similar to GADH.

We have developed a strawberry beverage in which D-glucose is completely fermented to GA or some other acids, yet fruit fructose is maintained as a natural sweetener (Cañete-Rodríguez et al., 2015, 2016). GA could be found naturally in fruit juices, honey, yogurt, bread, cottage cheese and meat. This acid gives a refreshing sour taste to wine and fruit juices and has the property of preventing bitterness in foodstuffs. In the food industry, GA is widely used as flavoring agent and for reducing absorption of fat products and is listed as a generally permitted food additive (E574) by the EFSA, and as a GRAS (Generally Recognized As Safe) additive by the US FDA (Ramachandran et al., 2006). Moreover, GA has been reported to have some beneficial effects on intestinal microbiota (Asano et al., 1994, 1997; Tsukahara et al., 2002) and it has limited toxicity. This low toxicity makes GA useful for food additives as one of the common counter ions for the administration of some metal cations (Zn, Ca, Na, K) or other chemicals (chlorhexidine). However, the equimolar conversion of D-glucose into GA and the high D-glucose concentrations in some fruits might recommend the reduction of GA by further oxidation. Therefore, the knowledge of the possible transformations of D-glucose into different metabolites would help control the levels of the different compounds in these transformed fruit beverages. In a previous study (Sainz et al., 2016), three natural AAB strains were selected for this GA fermentation using different media and conditions, but especially focusing on the strawberry process. Two of these strains belong to the *Gluconobacter* genus: *Gluconobacter japonicus* strain CECT 8443 isolated from grape must (Navarro et al., 2013) and *Gluconobacter oxydans* strain Po5 isolated from wine vinegar (Vegas et al., 2010). The other strain from *Acetobacter malorum* (CECT 7742) was the only strain isolated from strawberry vinegar (Hidalgo et al., 2013).

The aim of the present study was to compare the enzyme activities of the membrane-bound dehydrogenases responsible for D-glucose and GA oxidations in six strains of three different AAB species (selected strains from our collection and other strains from other culture collection strains). We wanted to analyze the effect of these enzyme activities on the production of the involved metabolites (GA, 2KGA and 5KGA) for better control of the production of these fermented beverages.

## Materials and Methods

### Microorganism and Culture Conditions

Two strains of each AAB species (*G. oxydans, G. japonicus and A. malorum*) were used in this study (**Table 1**). For the preparation of the inocula, these strains were previously grown for 24 h in 5 mL potato media (Matsushita and Ameyama, 1982) with shaking at 28°C. Experiments were performed in Erlenmeyer flasks of 500 mL with 100 mL media (30 g/L of D-glucose (Wako Pure Chem., Osaka, Japan), 40 g/L of D-fructose (Wako Pure Chem.), 5 g/L of polypeptone (Nihon Pharmaceutical Co., Ltd, Tokyo, Japan) and 5 g/L of yeast extract (Oriental Yeast Co., Ltd, Tokyo, Japan)) and inoculated with 1 mL of the corresponding strain grown in potato media. The experiment was carried out in triplicate, with shaking (200 rpm) at 28°C and sampled at 24, 48, and 96 h. Bacterial growth was measured by a Klett-Summerson photoelectric colorimeter with a red filter.

**Table 1:** Strains used in this study.

Species	Strain	Source	Reference
*Gluconobacter japonicus*	CECT 8443	Grape must	Navarro et al., 2013
	NBRC 3271^T^	*Myrica rubra* (Fruit)	Malimas et al., 2009
*Gluconobacter oxydans*	Po5	Vinegar	Vegas et al., 2010
	621 H	–	De Ley, 1961
*Acetobacter malorum*	CECT 7742^a^	Strawberry vinegar	Hidalgo et al., 2013
	NBRC 108912^T^	Rotting apple	Cleenwerck et al., 2002

*^T^Type strains. ^a^This strain has been incorrectly named CECT 7749 in previous studies (Hidalgo et al., 2013 and Sainz et al., 2016).*

### Preparation of Membrane Fraction

As explained previously, cells were harvested at 24, 48, and 96 h. The total volume (100 mL) was centrifuged for 5 min at 10.600 × *g*, and the cells were washed twice with 50 mM potassium phosphate buffer, pH 6.5 (1 g wet cells per 4–5 mL buffer). After washing, the pellets were stored for 24 h at 4°C and then resuspended in the same volume with the same buffer. The cell suspension was passed twice through a French pressure cell press (SIM AMINCO, Spectronic Instruments, Inc., Rochester, NY, USA) at 16.000 psi. Intact cells were removed with 10.000 × *g* for 10 min, and the supernatant was centrifuged at 100.000 × *g* for 60 min at 4°C. The resulting precipitate was resuspended in potassium phosphate buffer [1 M dipotassium phosphate (Wako Pure Chem.) and 1 M monopotassium phosphate (Wako Pure Chem.), pH 6.5] (20 mL buffer per 1 g pellet) and homogenized with the same buffer in a glass homogenizer. In the case of GLDH, 10 mM MES [2-(*N*-morpholino)ethanesulfonic acid, (Dojindo, Kumamoto, Japan)] – NaOH buffer was used. The resulting homogenate was considered the membrane fraction.

### Protein Determination

The protein concentration was determined by a modified Lowry method (Dulley and Grieve, 1975) using bovine serum albumin (Sigma, Tokyo, Japan) as the standard.

### Assays of Enzyme Activity

All enzymatic reactions were performed in triplicate and at 25°C. mGDH and GLDH were assayed in the presence of phenazine methosulfate (PMS) (Wako Pure Chem.) and 2,6-dichlorophenol indophenol (DCIP) (Wako Pure Chem.) as electron acceptors, as described by Matsushita et al. (1980). The 1 mL reaction mixture contains 50 mM potassium phosphate buffer (pH 6.5), 8 mM sodium azide (Wako Pure Chem.), 6.67 mM DCIP, 6 mM PMS, 100 mM D-glucose or glycerol (Wako Pure Chem.) as substrate and the membrane fraction. Some modifications were done for the GLDH assay; 10 mM acetate buffer [10 mM sodium acetate trihydrate (Wako Pure Chem) and acetic acid (Wako Pure Chem) (pH 6.0)] was used instead of potassium phosphate buffer. For the conversion of apo-enzyme to holo-enzyme, 3 mM calcium chloride anhydrate (Wako Pure Chem.) and 0.1 μM PQQ (Wako Pure Chem.) were added and incubated for 10 min in an ice bath. The enzyme activity was measured by the reduction of DCIP at 600 nm. One unit of enzyme activity was defined as the amount of enzyme catalyzing the oxidation of 1 μmol of substrate per min, which was calculated using the millimolar extinction coefficient of DCIP of 13.2 at pH 6.5 and of 11.13 at pH 6.0.

The enzyme activity of GADH and 2KGDH was measured according to Wood et al. (1962), using ferricyanide (Wako Pure Chem.) as an electron acceptor. The reaction mixture consists of 8 mM sodium azide, 100 mM ferricyanide, 100 mM GA (Sigma) or 2KGA (Sigma) as the substrate, the membrane fraction and McIlvaine buffer [a mixture of 0.1 M citric acid (Wako Pure Chem.) and 0.2 M disodium hydrogen phosphate (Wako Pure Chem.), pH 4.5] to a total volume of 1.0 mL. The reaction started with the addition of ferricyanide solution, and after 10 min, the reaction was stopped by adding 500 μL of ferric-Dupanol reagent (Wako Pure Chem.). Twenty minutes later, 3.5 mL of water was added, and after mixing well, the absorbance at 600 nm was measured by a UV-1700 PharmaSpec spectrophotometer (UV-1700 PharmaSpec, Shimadzu, Kyoto, Japan). Under these assay conditions, 4 absorbance units corresponded to 1 μmol of substrate oxidized.

### Determination of Substrates and Products by HPLC Analysis

All metabolites were analyzed using high performance liquid chromatography (HPLC – Shimadzu). D-Glucose and D-fructose were quantified on a Pb^2+^-loaded cation-exchange column (SUGAR SP0810, 8.0 mm I.D. × 300 mm L, Shodex, Showa denko KK, Kawasaki, Japan) at 80°C using distilled and deionized water as the mobile phase at a flow rate of 0.5 mL.min^-1^. Substances were detected with a refractive index detector. The retention times for D-glucose and D-fructose were 19.5 and 24.7 min, respectively. GA, 5KGA, and 2KGA were quantified on an ion-exclusion column (RSpak KC-811, 8.0 mm I.D. × 300 mm L, Shodex, Showa denko KK, Kawasaki, Japan) at 60°C using 0.1% (w/v) phosphoric acid as the mobile phase at a flow rate of 0.4 mL.min^-1^. Substances were detected with an UV detector (SPD-M20A, Shimadzu SPD-M20A) at 210 nm. The retention times of GA, 5KGA, and 2KGA were 18.8, 18.1, and 17.4 min, respectively.

### Primer Design and PCR Conditions

Genes coding for mGDH (*gcd*) and large subunits of GADH (*gnd*L), GLDH (*sld*A) and 2KGDH (*kgd*L) were partially amplified to confirm their presence. For this reason, the primers for these genes were designed using the program Primer3Plus (Untergasser et al., 2007) in each species using the sequences available in the GenBank database (**Table 2**). The amplification reaction was carried out in a total volume of 50 μL consisting of 1 μL of DNA solution, 5 μL of 10 X buffer, 3 μL of MgCl_2_, 200 μM each of the four dNTPs (Roche Diagnostic GmBh, Manheim, Germany), 0.4 μL of BSA (20 mg/mL), 4 μL of DMSO, 1 μL of each primer (10 pmol), and 0.4 μL of Taq Polymerase (Biotaq, Bioline – USA). The conditions of the PCR were as follows: initial denaturation at 94°C for 5 min, followed by 30 cycles of denaturing at 94°C for 1 min, annealing at 55°C or 60°C (depending on the primers) for 30 s, extension at 72°C for 1 min and a final extension at 72°C for 10 min and maintained at 4°C. The amplifications were performed in a Gene Amp PCR System 2700 (Applied Biosystems, Foster city, USA), and the PCR products were detected by electrophoresis gel on 1% agarose in 1X TBE buffer. The gels were stained with ethidium bromide and photographed.

**Table 2:** PCR pair primers used for gene amplification.

Strain	GenBank accession No.	Locus tag of gene sequences used for primer design	Gene	Product	Primer name	Primer sequence (fwd)	Primer sequence (rev)
*Gluconobacter japonicus* NBRC 3271	LHZK00000000	AD938_10885	*gcd*	Membrane-bound glucose dehydrogenase	*mgdh*	TGGTTTTCCCGGGTGATCTG	GTAGTAGTCCATCGTGCCCG
	LHZK00000000	AD938_08480	*gnd*L	Gluconate 2-dehydrogenase, large subunit	*gadh1*	TCCTGAGTGCGTTCCAGTTC	CGCTTTGGCAATGGGTTCAA
	LHZK00000000	AD938_03325	–	Gluconate 2-dehydrogenase, large subunit	*gadh2*	GGCCTATCCCTCGTCAATCG	TGCATAACCGCTGCAAAACC
	LHZK00000000	AD938_10275	*sld*A	Glycerol dehydrogenase large subunit SldA	*gldh1*	CGGGTGAAGAGAAGTGGGTC	GAGCTGGTCATACATCGGGG
	LHZK00000000	AD938_11075	*sld*A	Glycerol dehydrogenase large subunit SldA	*gldh2*	GGTAAGGAGATCTGGCGTCG	TGAAACTGCATTTTCCGCCG
*Gluconobacter oxydans* 621H	CP000009	GOX0265	*gcd*	Membrane-bound glucose dehydrogenase	*mgdh*	CTCGTGTACATCCCGATGGG	ACCACCCCACTCGAACATTC
	CP000009	GOX1231	*–*	Gluconate 2-dehydrogenase, large subunit	*gadh*	TATTGCAGCGGCTATGACTG	CATGGTCGAAATTCATGCTG
	CP000009	GOX0854	*sld*A	Glycerol dehydrogenase large subunit SldA	*gldh*	GCGACGGGTAAGGAGATCTG	TTTCTTCAGGGCTACGCAGG
*Gluconobacter oxydans* NBRC 3293	AB985494	–	*kgd*L	Large subunit of 2-keto-D-gluconate dehydrogenase	*2kgdh*	GGAAAACTGGCGCAACATGTCG	CCCGAACGGGATCATGTC
*Acetobacter malorum* strain DmCS_005	JOJU00000000	AmDm5_2097	*–*	Membrane-bound glucose dehydrogenase	*mgdh*	ATGTTTGAATGGGGCGGTCT	CGTCATACGCCCGGATGTAA
	JOJU00000000	AmDm5_1995	*–*	Gluconate 2-dehydrogenase, large subunit	*gadh*	CGGGTGAAGCCTATACGGTC	AGAATGACAAGTCCGGCAGG
	JOJU00000000	AmDm5_0421	*kgd*L	Large subunit of 2-keto-D-gluconate dehydrogenase	*2kgdh*	ACCTGCCGTCAGACTTTGAG	ATACAATGCGCGGCAATCAC

### Sequence Alignment and Phylogenetic Tree Construction

The nucleotide sequences of genes *gcd* and *gnd*L of the natural strains used in this study have been sequenced and deposited in the GenBank Database with the following accession numbers: *G. oxydans* Po5 (KU896941, KU896943), *A. malorum* CECT 7742 (Amal_02000, Amal_01874) and *G. japonicus* CECT 8443 (A0J51_02827, A0J51_00901). The *sld*A gene sequence was not found in *A. malorum* and the corresponding sequences for *Gluconobacter* species were A0J51_00428 and A0J51_00622 for *G. japonicus* and KU896942 for *G. oxydans*. These sequences were compared with the sequences from other genera and species available in GenBank database for the phylogenetic analyses. The sequence alignment was performed using the nucleotide sequence with the MUSCLE 3.8.31 software (Edgar, 2004a,b). The poorly aligned regions were removed using the Gblocks 0.91b program (Castresana, 2000; Talavera and Castresana, 2007).

The phylogenetic tree was reconstructed using the maximum likelihood method implemented in the PhyML program (v3.1/3.0 aLRT). The HKY85 substitution model was selected assuming an estimated proportion of invariant sites (of 0.248) and 4 gamma-distributed rate categories to account for rate heterogeneity across sites. The gamma shape parameter was estimated directly from the data (γ = 0.770). Reliability for internal branch was assessed using the aLRT test (SH-Like). The tree rendering was performed with the TreeDyn 198.3 graphical editor^1^ (Dereeper et al., 2008, 2010).

## Results

**FIGURE 1:**
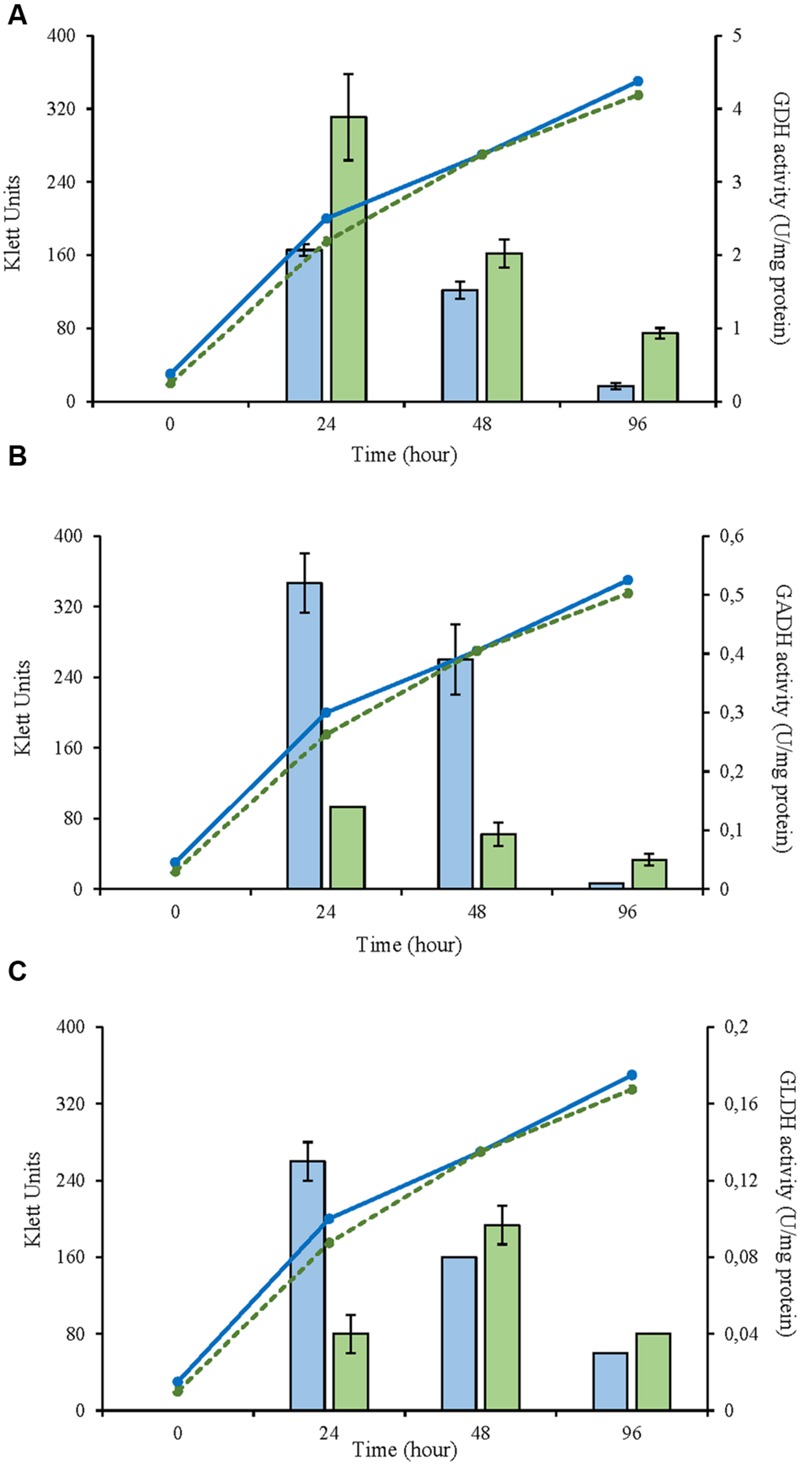
**Enzyme activity and growth (expressed in Klett units) of two *Gluconobacter japonicus* strains at three different growth stages (24, 48, and 96 h). (A)** Glucose dehydrogenase activity (mGDH); **(B)** Gluconate dehydrogenase activity (GADH); **(C)** Glycerol dehydrogenase activity (GLDH). Enzyme activity represented in bars: NBRC 3271 (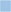), CECT 8443 (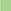) and cell growth with lines: NBRC 3271 (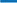); CECT 8443 (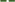).

**FIGURE 2:**
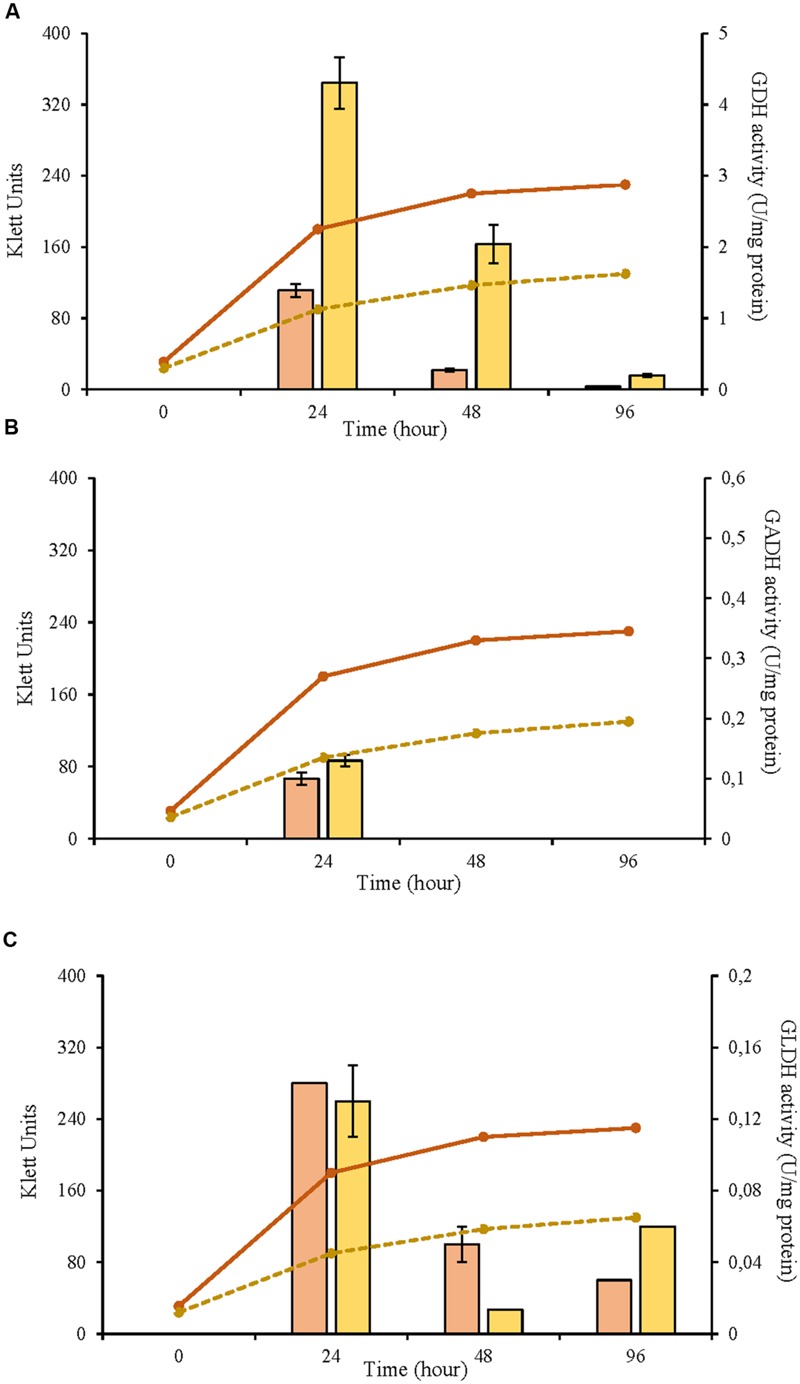
**Enzyme activity and growth (expressed in Klett units) of two *Gluconobacter oxydans* strains at three different growth stages (24, 48, and 96 h). (A)** Glucose dehydrogenase activity (mGDH); **(B)** Gluconate dehydrogenase activity (GADH); **(C)** Glycerol dehydrogenase activity (GLDH); Enzyme activity represented in bars: 621H (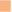), Po5 (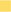) and cell growth with lines: 621H (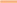); Po5 (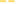).

**FIGURE 3:**
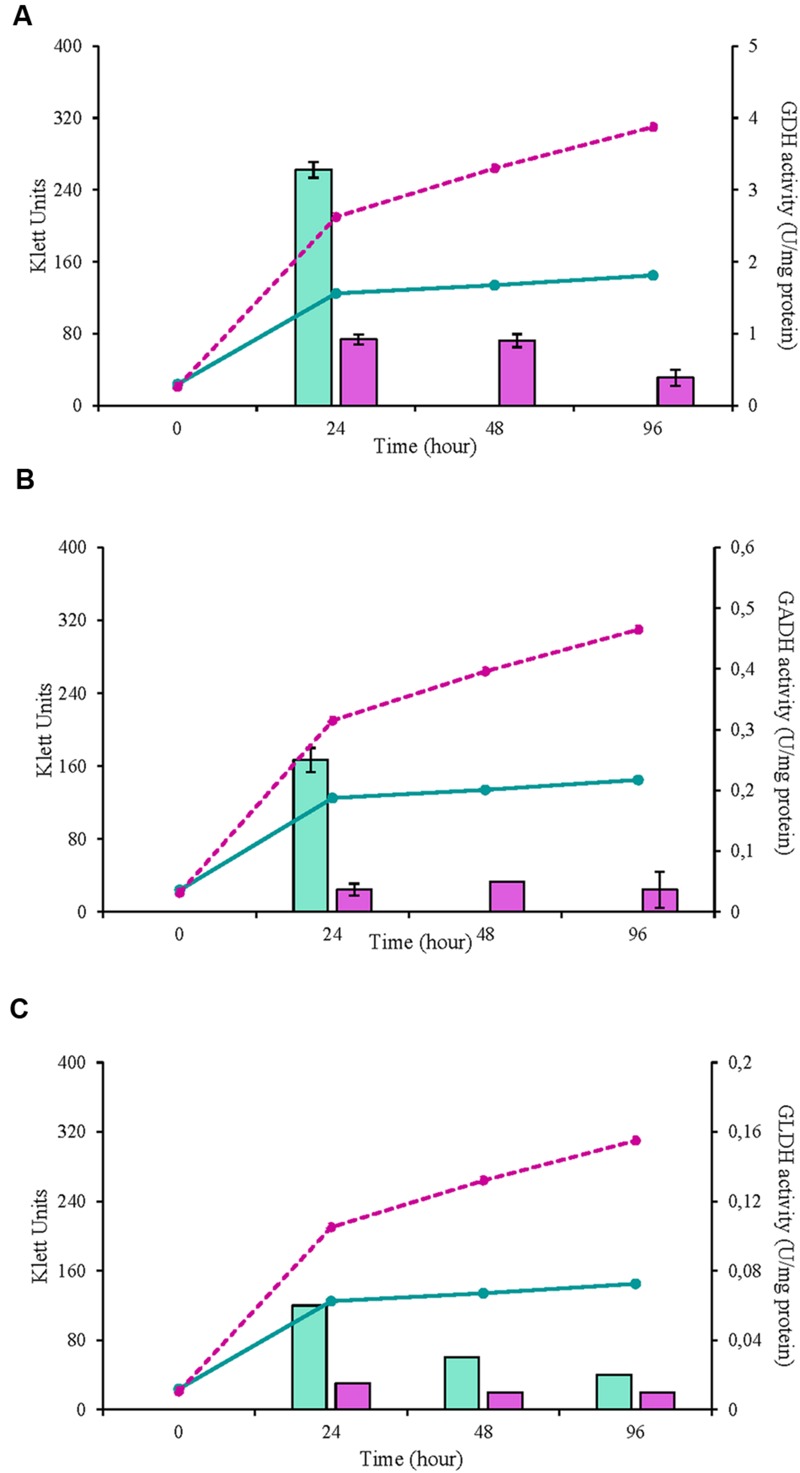
**Enzyme activity and growth (expressed in Klett units) of two *Acetobacter malorum* strains at three different growth stages (24, 48, and 96 h). (A)** Glucose dehydrogenase activity (mGDH); **(B)** Gluconate dehydrogenase activity (GADH); **(C)** Glycerol dehydrogenase activity (GLDH). Enzyme activity represented in bars: NBRC 108912 (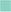), CECT 7742 (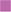) and cell growth with lines: NBRC 108912 (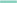); CECT 7742 (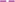).

In this study, three selected AAB strains, belonging to *G. japonicus, G*. *oxydans*, and *A. malorum* species, isolated from vinegar or fruit were examined together with their corresponding culture collection strains in terms of growth, enzyme activities involved in the D-glucose oxidation, and metabolites produced from oxidation. For the *G. japonicus* species, both the isolated and the type culture strains showed very similar growth (**Figure 1**), achieving a high population at the end of the experiment (320 Klett units at 96 h), without reaching the stationary phase. Both strains presented a high mGDH activity and a similar evolution over time (**Figure 1A**). In both cases, mGDH activity is maximal at 24 h, although strain CECT 8443 exhibited twice the activity of NBRC 3271, and the activity decreased afterward. In relation to GADH, both strains presented similar behavior, showing the highest activity at 24 h (**Figure 1B**). However, strain NBRC 3271 had fourfold higher activity than CECT 8443 during the first 48 h followed by a sharp decline, resulting in a GADH activity being practically absent at 96 h. Instead, the GLDH activity in these strains presented low activity (lower than 0.15 U/mg protein in all the cases) and behaved differently from each other (**Figure 1C**). Strain NBRC 3271 presented the highest activity at 24 h and decreased afterward, whereas strain CECT 8443 exhibited the highest activity at 48 h. The *G. oxydans* strains (**Figure 2**), although they had a very similar initial population, presented huge differences in their growth, mainly due to the first 24 h, when strain 621H achieved twice the population of Po5. After this moment, the evolution in both strains was very similar, showing slower growth and entry in the stationary phase. Similarly, *G. oxydans* Po5 presented the highest activity of mGDH (**Figure 2A**) at 24 h, when maximal activity was reached, which was three times higher than in 621H. Then, a clear decrease of the activity was observed in both cases. The GADH activity was only detected at 24 h in both *G. oxydans* strains, with similar values (∼0.1 U/mg protein) (**Figure 2B**). In the case of GLDH, *G. oxydans* strains showed similar activity at 24 h (**Figure 2C**), later presenting a reduction in the activity. However, in strain Po5, this decrease was more pronounced at 48 h, but an upturn of activity was observed at the end (96 h). Finally, *A. malorum* strains presented a similar evolution of *G. oxydans* strains, although in this case the wild strain (CECT 7742) grew better than the type strain (NBRC 108912) (**Figure 3**). In this case, the difference in growth between both strains (∼90 Klett units) was mainly observed during the first 24 h. After these 24 h, CECT 7742 showed some growth, although with a lower rate, whereas the type strain was not growing. Strain NBRC 108912 showed a very high mGDH activity at 24 h; however, no activity was detected afterward (**Figure 3A**). In contrast, CECT 7742 presented less activity but maintained the activity over time (1 U/mg protein at 24 and 48 h and half at 96 h). The activity of GADH presented similar evolution as mGDH, although with much lower values. In strain NBRC 108912, GADH activity was only detected at 24 h, and with the highest value, whereas CECT 7742 presented a low and constant activity over time (**Figure 3B**). Finally, low GLDH activity was observed in both *A. malorum* strains (**Figure 3C**), although the activity was higher in NBRC 108912. In CECT 7742, residual activity was observed in all the points studied.

The activity of 2KGDH was also studied in all the strains. However, no activity was detected in any of these strains (**Table 3**).

**Table 3:** Results of PCR analyses of *gcd, gnd*L, *sld*A, and *kgd*L genes, and of enzyme activity for the six tested acetic acid bacteria strains.

		*gcd*		*gnd*L		*sld*A		*kgd*L	
Species	Strain	Activity	Gene	Activity	Gene	Activity	Gene	Activity	Gene
*Gluconobacter japonicus*	CECT 8443	+	+	+	+^a^	+	+^b^	-	n.d
	NBRC 3271	+	+	+	+	+	+	-	n.d
*Gluconobacter oxydans*	Po5	+	+	+	+	+	-	-	-
	621H	+	+	+	+	+	+	-	-
*Acetobactermalorum*	CECT 7742	+	+	+	+	+	n.d	-	+
	NBRC 108912	+	+	+	+	+	n.d	-	+

*n.d, not determined. ^a^Amplified only by primers *gadh1* (**Table 2**). ^b^Amplified only by primers *gldh2* (**Table 2**).*

**FIGURE 4:**
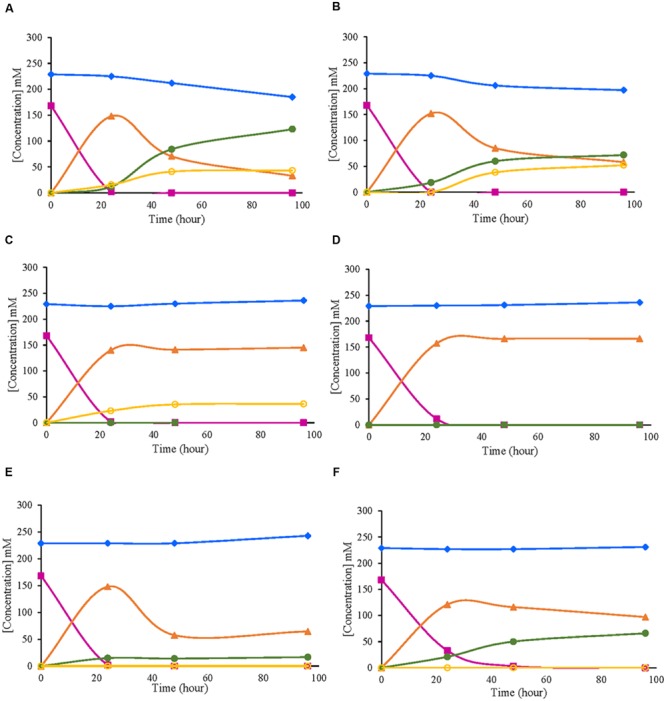
**Evolution of metabolites derived from D-glucose oxidation by six different strains of acetic acid bacteria. (A)** *Gluconobacter japonicus* NBRC 3271; **(B)** *Gluconobacter japonicus* CECT 8443; **(C)** *Gluconobacter oxydans* 621H; **(D)** *Gluconobacter oxydans* Po5: **(E)** *Acetobacter malorum* NBRC 108912; **(F)** *Acetobacter malorum* CECT 7742. D-glucose (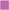); D-fructose (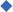); D-gluconic acid (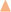); 2-keto-D-gluconic acid (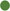); 5-keto-D-gluconic acid (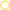).

In the tested strains, evolution of the metabolites derived from D-glucose oxidation was analyzed at the same time points when the enzymatic activity was measured (24, 48, and 96 h). Similar patterns between strains of the same species were obtained according to the consumption and production of the metabolites studied. In *G. japonicus* and *G. oxydans* strains, D-glucose was totally exhausted at 24 h, when the maximum accumulation of GA in the medium was observed (**Figures 4A,B**). Moreover, in *G. japonicus* strains, the depletion of D-glucose appeared to be correlated with the beginning of the oxidation of GA, resulting in the accumulation of 2KGA and 5KGA in the medium. Unlike what happened in strain NBRC 3271, where the initial accumulation of both keto-D-gluconates was similar, in CECT 8443, (**Figure 4B**) the accumulation of 2KGA occurred before, not detecting 5KGA until 48 h. Both *G. japonicus* strains accumulated more 2KGA than 5KGA, although this difference was really remarkable in the type strain, in which the 2KGA concentration was three times higher than 5KGA. The consumption of GA was not observed in *G. oxydans* strains, and it mostly accumulated in the medium (**Figures 4C,D**). However, strain 621H produced 5KGA in similar amounts to those obtained with *G. japonicus* NBRC 3271. This accumulation of 5KGA compensated for the lower accumulation of GA in this strain 621H compared with Po5. In the *A. malorum* strains, only NBRC 108912 (**Figure 4E**) consumed all D-glucose at the first 24 h, whereas CECT 7742 (**Figure 4F**) consumed the substrate by 48 h. Moreover, after the maximal accumulation of GA (24 h in both *A. malorum* strains), 56% of GA produced was further oxidized in NBRC 108912, whereas only 19% was further oxidized in CECT 7742. CECT 7742 accumulated four times more 2KGA than NBRC 108912.

The presence of the genes coding for the measured enzymes (*gcd, gnd*L, *sld*A and *kgd*L) was confirmed by the amplification of a fragment of these genes. All primer sequences were designed from already available genome sequences of these three AAB species (see **Table 2**). In strain NBRC 3271, because two set of genes for GADH and GLDH are present, two sets of primers (*gadh1* and *gadh2*; *gldh1* and *gldh2*) were designed (**Table 2**). As expected, the presence of the genes for mGDH (*gcd*) and GADH (*gndL*) was confirmed for all the strains (**Table 3**). However, in strain *G. japonicus* CECT 8443, only one set of primers (*gadh1*) worked for the amplification of *gndL*, and specific primers (*mgdh*) for *gcd* of NBRC 3271 did not work, although amplification was achieved with *G. oxydans* primers. In the case of the GLDH gene, no amplification was obtained in *G. oxydans* Po5 despite presenting activity, and in *G. japonicus* CECT 8443, as in GADH genes, only one set of primers (*gldh2*) worked. Finally, the 2KGDH gene (*kgdL*) was amplified only in *A. malorum* strains, although activity was not detected.

**FIGURE 5:**
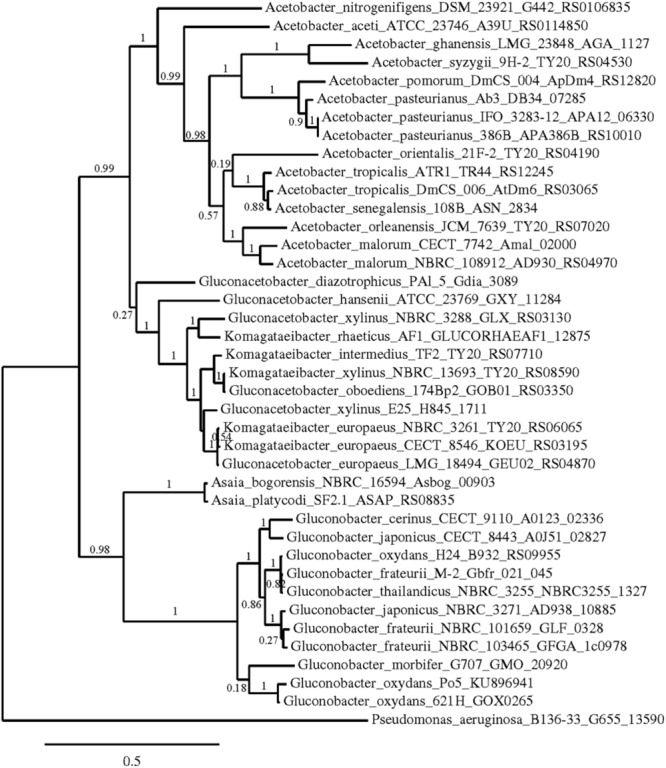
**Phylogenetic relationship of *gcd* gene in different species of acetic acid bacteria.** The entries of different genotypes include the accession numbers of the GeneBank database sequence. The *gcd* sequence of *Pseudomonas aeruginosa* B136-33 was used as outgroup. The numbers indicate the branch support values.

**FIGURE 6:**
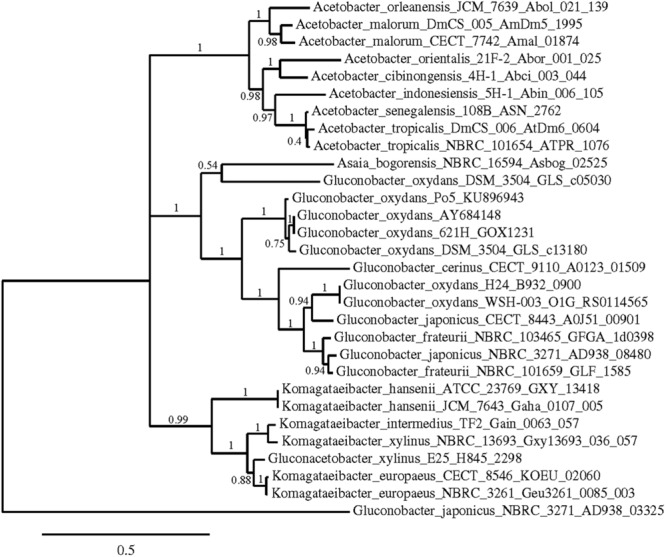
**Phylogenetic relationship of *gnd*L gene in different species of acetic acid bacteria.** The entries of different genotypes include the accession numbers of the GeneBank database sequence. The sequence of one of the genes coding for GADH in *Gluconobacter japonicus* NBRC 3271 (AD938_03325) was used as outgroup. The numbers indicate the branch support values.

**FIGURE 7:**
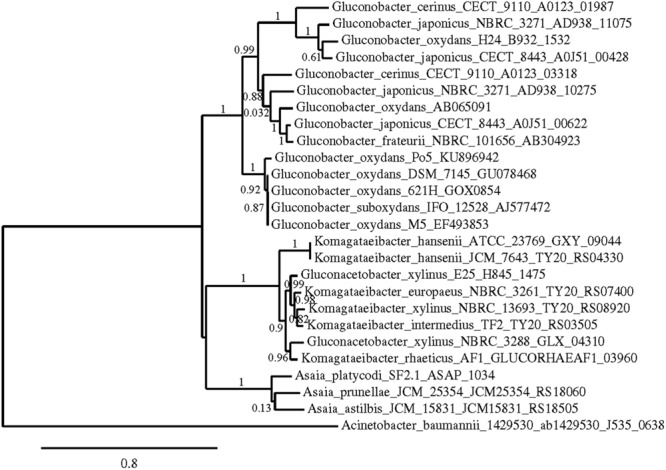
**Phylogenetic relationship of *sld*A gene in different species of acetic acid bacteria.** The entries of different genotypes include the accession numbers of the GeneBank database sequence. The *sld*A sequence of *Acinetobacter baumannii* 1429530 was used as outgroup. The numbers indicate the branch support values.

Phylogenetic trees were constructed using the nucleotide sequences of these genes in these strains in comparison with sequences available in the GenBank Database (**Figures 5–7**). In all cases, AAB genera were clustered separately according to these gene sequences. In the case of the mGDH gene (**Figure 5**), two branches were clearly observed; one branch included the *Komagataeibacter* and *Acetobacter* species and the branch included the *Gluconobacter* and *Asaia* species. In the *Acetobacter* branch, both *A. malorum* enzymes grouped with *A. orleanensis, A. senegalensis* and *A. tropicalis* and were separated from those of *A. pasteurianus, A. pomorum, A. ghanensis, A. syzygii* and *A. aceti*. In the case of the *Gluconobacter* cluster, different species were mixed, and no specific groupings were observed. Our *G. oxydans* enzymes grouped together while our *G. japonicus* strains were separated in different subclusters. In the case of the GADH gene (**Figure 6**), the sequence of one of the genes (the one that was amplified with the set of primers *gadh2*) of *G. japonicus* NBRC 3271 appeared as outgroup. The other sequences were grouped in three clusters, one for *Acetobacter* species, another for *Komagataeibacter* species and the last one for *Gluconobacter* species together with one *Asaia* sequence. As in the mGDH tree, the two *A. malorum* enzymes grouped together. In the *Gluconobacter* cluster, there were two branches; one branch consisted of *Asaia bogorensis* sequence and *gndL* (which was amplified with set of primers *gadh2*) of a strain of *G. oxydans* (DSM 3504). All other sequences grouped together in a common branch. Finally, in the GLDH gene (*sldA*) tree (**Figure 7**), the sequences were grouped in two clusters; one branch included *Gluconobacter* sequences, and the other branch included *Komagataeibacter* and *Asaia* sequences. Unlike the other genes, in this case, the *Asaia* sequences clustered with *Komagataeibacter* but not with *Gluconobacter*. No *Acetobacter* sequences have been included because this gene has not been described in this genus. In the *Gluconobacter* cluster, three different groups were clearly defined; one cluster was basically *G. oxydans* sequences, while in the other two clusters, the two homologous GLDH genes (that were amplified by the primer sets of *gldh1* and *gldh2*) of *G. japonicus* grouped separately.

## Discussion

In a previous study (Sainz et al., 2016), we selected three strains of AAB capable of oxidizing D-glucose to GA without consuming D-fructose in a puree of strawberry with the aim of developing a new attractive fermented beverage for consumers preserving the fruit natural sweetness. The following three main requirements were decisive for the selection of these strains: a high production of GA, total consumption of D-glucose and minimal oxidation of D-fructose. Because the consumption of D-glucose and D-fructose and the production of GA were observed to be dependent on the strain and the media, the strains were mainly selected based on their behavior in strawberry puree. The selected strains were *G. japonicus* CECT 8443, *G. oxydans* Po5 and *A. malorum* CECT 7742, depending on the desired final product (final concentration of GA and keto-D-gluconates). Understanding the differences in the production of these compounds in these strains could help control the beverage composition in a more effective and reproducible way.

It has been extensively described that AAB present high numbers of membrane-bound dehydrogenases, classified as quinoproteins and flavoproteins – cytochrome *c* complex, involved in incomplete oxidation of sugars and alcohols to produce the corresponding sugar acids which are accumulated in the medium (Matsushita et al., 1994, 2004; Adachi et al., 2007). This feature is essential for industrial applications of these organisms (Meyer et al., 2013). In our study, different AAB strains were collected at different growth phases, and the activity of membrane-bound dehydrogenases involved in D-glucose oxidation and the accumulation of corresponding metabolites were studied.

According to the growth of the strains, differences in the maximal population were observed both among species and between strains within the same species, reaching in some cases double the population size. Only *G. japonicus* strains showed identical growth, achieving the highest population of all the studied species. A low biomass formation has been associated with high oxidation rates (Elfari et al., 2005); the more biomass produced, the less D-glucose used for product formation. Krajewski et al. (2010) explained this low biomass when D-glucose is used as carbon source because most D-glucose is metabolized rapidly to GA and its derivatives in the periplasm, and therefore, it could not be used for biomass formation, just for the generation of proton motive force. In our case, no important differences were observed between the species or strains because D-glucose was completely depleted in all the cases, and the maximal accumulation of GA in the medium was similar, except for strain CECT 7742 belonging to *A. malorum* that accumulated approximately 30 mM less of GA. In this case, the lower accumulation of GA was compensated by a high concentration of 2KGA. Therefore, there was no correlation between low growth and high GA and keto-D-gluconates production, likely because growth in all cases was low, suggesting that the amount of D-glucose used for biomass was only a minor part of the initial D-glucose, and the differences observed in growth were not reflected in metabolite production. This low growth confirms that the oxidation of D-glucose to GA and keto-D-gluconates has a negative effect on the growth rate and the growth yield as stated by Krajewski et al. (2010) for *G. oxydans*.

All strains accumulated GA in the medium, being maximal at 24 h, in parallel with the maximal activity of mGDH. This high enzyme activity at the late exponential phase and its subsequent decrease agree with the findings of Matsushita et al. (1980), who described that mGDH activity reached the maximum activity in the mid-to late exponential phase of cultivation and then decreased with progress of growth. Ameyama et al. (1981) observed that the higher formation of this enzyme was achieved at the late exponential phase, between 24 and 30 h, depending on the fermenter used, when AAB grew on a medium containing D-glucose, glycerol and sodium-D-gluconate. The activity levels obtained in this study are consistent with those found in the literature (Ameyama et al., 1981; Matsushita et al., 1987, 1989; Meyer et al., 2013), showing similar or even higher values. Important differences in mGDH activity among strains were observed, and these differences were especially relevant in the case of selected *Gluconobacter* strains with a high activity along the process in comparison to culture collection strains. However, in practically all cases, similar GA concentrations were detected, probably due to a limitation of substrate (D-glucose) in the media. Moreover, as the first sampling point of activity (24 h) already showed the highest enzyme activity, a similar evolution over time was observed in the enzyme activities between strains.

Regarding GADH, all our tested strains showed GADH activity. Shinagawa et al. (1976) previously reported this activity in the cell free extracts in strains of *Gluconobacter* and *Acetobacter* together with strains from other bacteria (*Pseudomonas aeruginosa, Klebsiella pneumoniae* and *Serratia marcescens*). In our study, *G. japonicus* NBRC 3271 presented the highest activity of GADH and the highest accumulation of 2KGA, whereas *G. oxydans* strains did not accumulate any 2KGA despite presenting small activity only at 24 h. Strains from different species of the *Gluconobacter* genus are reported to accumulate high concentrations of 2KGA and/or 5KGA from D-glucose or GA without any appreciable assimilation into cells (Sievers and Swings, 2005). Moreover, a sequential accumulation of GA and keto-D-gluconates during the growth of *G. oxydans* 621H and other *Gluconobacter* species on D-glucose media with controlled pH has been described (Weenk et al., 1984), which is in agreement with our results. However, Levering et al. (1988) showed that *G. oxydans* 621H growing in yeast extract medium containing 50 mM D-glucose was able to oxidize quantitatively D-glucose to GA, without the production of 2KGA and 5KGA, similar to our observations with strain Po5. This lack of keto-D-gluconates synthesis in some strains has been associated with the fact that during the first phase of growth on D-glucose in batch cultures, the oxidation of D-glucose by mGDH was so rapid that the respiratory chain becomes saturated. For this reason, the ubiquinone was unable to accept electrons from GADH, resulting in the impossibility of oxidizing the GA in these conditions (Levering et al., 1988). Therefore, strain Po5, with high production of GA but no accumulation of keto-D-gluconates, appeared to be the best strain to obtain and maintain high concentrations of GA in the fermented beverage. In our previous study (Sainz et al., 2016), we tested different media, and this strain was the strain with the highest production of GA and the lowest production of keto-D-gluconates. Diverse studies in *Gluconobacter* strains showed differences in the rate of 2KGA or 5KGA from D-glucose (Weenk et al., 1984; Silberbach et al., 2003; Herrmann et al., 2004; Elfari et al., 2005). The individual product yields vary among different strains and depend also on the media and on the particular conditions used for cultivation (Asai, 1968; Olijve and Kok, 1979). GLDH and GADH enzymes compete for the oxidation of GA; therefore, selective expression of either dehydrogenase could increase the production of either of the keto-D-gluconates (Matsushita et al., 2003; Elfari et al., 2005). In our conditions, only *G. japonicus* strains were able to accumulate both keto-D-gluconates. These strains present two genes for GLDH, and strain NBRC 3271 also presents two genes for GADH. However, strain CECT 8443 has only one gene for GADH with a sequence similar to the gene, which was amplified with primers *gadh1* of strain NBRC3271.

*Gluconobacter oxydans* 621H only accumulated 5KGA. In other studies and culture conditions, 621H exhibited different keto-D-gluconate synthesis profiles, varying from the accumulation of both keto-D-gluconates (Weenk et al., 1984) or no keto-D-gluconates synthesis, confirming that culture conditions are essential for the synthesis of these compounds. The other strain belonging to *G. oxydans*, Po5, did not accumulate any keto-D-gluconate, despite having a similar GLDH activity to 621H. A lack of amplification of the GLDH gene (*sldA*) was observed in this strain (Po5). The 621H *sldA* sequence was used for the design of the primers, and although this gene sequence in both strains is similar (>96%), there are some nucleotide differences in the region where the reverse primer hybridized (results not shown). In *G. japonicus*, strain NBRC 3271 showed the highest GLDH activity at 24 h and after a decrease, although the 5KGA concentration was increasing until 48 h. Instead, in CECT 8443, the increase in GLDH activity between 24 and 48 h was correlated with the increase in the 5KGA accumulation. *A. malorum* strains presented both activities (GADH and GLDH), but no accumulation of 5KGA was detected. A lack of 5KGA synthesis was expected according to the *A. malorum* description (synthesis of 2KGA and lack of 5KGA synthesis) (Cleenwerck et al., 2002). However, the activity detected in this study together with the accumulation of this compound by strain CECT 7742 in a previous study (Sainz et al., 2016) appear to confirm that this species or some strains belonging to this species are able to synthesize 5KGA. It has to be emphasized that this previous production of 5KGA was observed in different medium conditions. The absence of the *sldA* sequence in the *A. malorum* genome appears to suggest the possibility that other enzymes for the synthesis of this compound are used. Furthermore, strain NBRC 108912 showed a high decrease in the GA concentration that cannot be only accounted for the oxidation to 2KGA. Nevertheless, the products of D-glucose oxidation have been reported to be assimilated by cytoplasmic reductases during the stationary phase, and then introduced to the pentose-phosphate pathway to produce cell biomass (Saichana et al., 2015). However, this would have as consequence a second phase of growth that was not observed in our case.

A phylogenetic study using the sequences of these three key enzymes for D-glucose oxidation in AAB was performed; in all the cases, trees that showed clear clusters according to the genus were obtained. Gene *gcd* was the one with more sequences available in the GenBank database, allowing for a more reliable study. Based on the *gcd* sequences, the *Acetobacter* and *Komagataeibacter* species seemed to be more closely related, and *Gluconobacter* was more related to *Asaia*, which is different to the findings obtained using the 16S rRNA gene sequence (Yamada et al., 2012). However, this difference should not be surprising because the D-glucose metabolism of these two genera is closer than in the other genera, which have a higher preference for other substrates, such as ethanol. The *Gluconobacter* and *Asaia* genera were reported to develop better in media enriched with sugar (Raspor and Goranovic, 2008), with high oxidation activity of sugar and sugar alcohols (D-glucose, GA, D-sorbitol, and glycerol). In addition, Matsutani et al. (2011) claimed that the *Acetobacter* and *Komagataeibacter* species are more closely related to each other than *Gluconobacter* by whole genome level phylogenetic analysis. Therefore, our results agree with this previous work.

For our results, the concentrations of D-glucose and GA show an effective, almost equimolar conversion, which takes place during the first 24 h and is likely to the end of the exponential phase of growth (Matsushita et al., 1980; Ameyama et al., 1981). At this time, the mGDH activity is the highest during the studied period. The absence of the main substrate makes its activity unnecessary and therefore declines afterward. It could be assumed that during the first 24 h, the high activity of this enzyme accounts for the full transformation of D-glucose into GA, which occurs in all the species and strains observed. However, the transformation of GA is heavily dependent on the species and the strain (Asai, 1968; Olijve and Kok, 1979; Weenk et al., 1984). Regardless of the presence of the activities of GADH and GLDH in *G. oxydans* natural strain Po5, no further oxidation of GA to keto-D-gluconates was observed. In fact, no production of 5-KGA was detected despite the high activity of GLDH in *G. oxydans*, showing a lack of correlation between the activity and products that could be explained by the lack of specificity of this enzyme (Matsushita et al., 2003) Instead, the absence of GADH activity after 24 h correlated with the lack of 2KGA production in both *G. oxydans* strains. In *G. japonicus* and *A. malorum*, the production of 2KGA was always observed, although no correlation could be found between the activity and the products. However, when activity of GADH was detected after 24 h, albeit it was low, important accumulation of 2KGA in the medium was observed (higher than 50 mM). Comparing between the three selected strains, important differences were observed at the activity level of these enzymes. Both *Gluconobacter* strains (CECT 8443 and Po5) presented a very high activity of mGDH at 24 h, with a further decrease and low activity in the GADH and GLDH but with changes overtime, whereas CECT 7742 presented in the three enzymes a low activity but maintained practically constant throughout all the time.

The possible use of these different strains and species for the production of different concentrations of GA and its derivatives could be achieved through the thorough knowledge of the activity and the expression of the enzymes. However, our results also indicate that the conditions of the process and the composition of the medium are crucial to the final composition of the product because important differences were observed in the synthesis profile of these strains in different media or conditions (Sainz et al., 2016). Therefore, a next step should be the analysis of the expression of these genes (especially mGDH and GADH) in different conditions to fully understand and control the process of the oxidation of D-glucose by AAB.

## Author Contributions

FS, MJT, KM, and AM Design and draft the work, interpret the data and wrote the final version. FS and MJT performed and analyzed the experimental work. MM, NK, and TY helped in the experimental work, revised critically the manuscript and approved the final version.

## Conflict of Interest Statement

The authors declare that the research was conducted in the absence of any commercial or financial relationships that could be construed as a potential conflict of interest.

## Footnotes

1 http://www.phylogeny.fr
